# Circulating Mitochondrial-Derived Vesicles, Inflammatory Biomarkers and Amino Acids in Older Adults With Physical Frailty and Sarcopenia: A Preliminary BIOSPHERE Multi-Marker Study Using Sequential and Orthogonalized Covariance Selection – Linear Discriminant Analysis

**DOI:** 10.3389/fcell.2020.564417

**Published:** 2020-09-22

**Authors:** Emanuele Marzetti, Flora Guerra, Riccardo Calvani, Federico Marini, Alessandra Biancolillo, Jacopo Gervasoni, Aniello Primiano, Hélio José Coelho-Júnior, Francesco Landi, Roberto Bernabei, Cecilia Bucci, Anna Picca

**Affiliations:** ^1^Fondazione Policlinico Universitario “Agostino Gemelli” IRCCS, Rome, Italy; ^2^Università Cattolica del Sacro Cuore, Rome, Italy; ^3^Department of Biological and Environmental Sciences and Technologies, Università del Salento, Lecce, Italy; ^4^Department of Chemistry, Sapienza Università di Roma, Rome, Italy; ^5^Department of Physical and Chemical Sciences, Università degli Studi dell'Aquila, L'Aquila, Italy

**Keywords:** aging, biomarkers, cytokines, extracellular vesicles, geroscience, metabolomics, mitochondrial dysfunction, mitochondrial quality control

## Abstract

Physical frailty and sarcopenia (PF&S) is a prototypical geriatric condition characterized by reduced physical function and low muscle mass. The multifaceted pathophysiology of this condition recapitulates all hallmarks of aging making the identification of specific biomarkers challenging. In the present study, we explored the relationship among three processes that are thought to be involved in PF&S (i.e., systemic inflammation, amino acid dysmetabolism, and mitochondrial dysfunction). We took advantage of the well-characterized cohort of older adults recruited in the “BIOmarkers associated with Sarcopenia and Physical frailty in EldeRly pErsons” (BIOSPHERE) study to preliminarily combine in a multi-platform analytical approach inflammatory biomolecules, amino acids and derivatives, and mitochondrial-derived vesicle (MDV) cargo molecules to evaluate their performance as possible biomarkers for PF&S. Eleven older adults aged 70 years and older with PF&S and 10 non-sarcopenic non-frail controls were included in the analysis based on the availability of the three categories of biomolecules. A sequential and orthogonalized covariance selection—linear discriminant analysis (SO-CovSel–LDA) approach was used for biomarkers selection. Of the 75 analytes assayed, 16 had concentrations below the detection limit. Within the remaining 59 biomolecules, So-CovSel–LDA selected a set comprising two amino acids (phosphoethanolamine and tryptophan), two cytokines (interleukin 1 receptor antagonist and macrophage inflammatory protein 1β), and MDV-derived nicotinamide adenine dinucleotide reduced form:ubiquinone oxidoreductase subunit S3 as the best predictors for discriminating older people with and without PF&S. The evaluation of these biomarkers in larger cohorts and their changes over time or in response to interventions may unveil specific pathogenetic pathways of PF&S and identify new biological targets for drug development.

## Introduction

Sarcopenia is the progressive decline in muscle mass and strength that accompanies aging (Marzetti et al., 2017). This condition exposes older adults to a high risk of negative health-related events, including disability, loss of independence, institutionalization, and death (Marzetti et al., 2017). The public health relevance of sarcopenia is widely recognized and so is the need for effective preventive and therapeutic interventions (Beaudart et al., 2014). Yet, the heterogeneous phenotypic presentation of sarcopenia, the insufficient understanding of its pathophysiology, and the frequent superimposition of other age-related conditions have hampered the study of sarcopenia as a standalone phenomenon (Calvani et al., 2018a). This impasse is also reflected by the lack of a unique operational definition of sarcopenia (Landi et al., 2018) and clinically meaningful biomarkers (Calvani et al., 2017). In this scenario, the recently defined “physical frailty and sarcopenia” (PF&S) syndrome has marked a major step forward for the clinical and regulatory recognition of the condition (Cesari et al., 2017).

When digging into the pathways and processes involved in PF&S pathophysiology, several factors spanning from muscle-specific mitochondrial dysfunction to systemic changes (e.g., inflammation, amino acid dysmetabolism) have been pinpointed (Marzetti et al., 2016, 2019; Picca et al., 2017a; Calvani et al., 2018b). Whether these processes share common roots and how cell-based alterations spread and are sensed at the systemic level are presently unknown. Small extracellular vesicle (sEVs) of mitochondrial origin, termed mitochondrial-derived vesicles (MDVs), have recently been proposed as possible shuttles across biological systems (Picca et al., 2020a). However, little is known about their complex regulatory network.

The pathophysiology of PF&S recapitulates all hallmarks of aging (López-Otín et al., 2013; Sierra, 2016; Justice et al., 2018). Hence, PF&S is considered to be a prototypical geroscience conditions for which a strong interdependence and non-linear relationships between biomarkers may be envisioned (Cohen et al., 2018). In such a scenario, the analysis of single pathways enlightening discrete aspects of the condition and, thus, setting aside its multifaceted nature, might neglect relevant information (Cohen et al., 2018; Justice et al., 2018). This limitation could be overcome through the adoption of multivariate analytical strategies that enable the exploitation of more comprehensive, multi-platform datasets (Calvani et al., 2015). In the present preliminary study, we took advantage of the well-characterized cohort of older adults recruited in the “BIOmarkers associated with Sarcopenia and Physical frailty in EldeRly pErsons” (BIOSPHERE) study (Calvani et al., 2018b,c; Marzetti et al., 2019; Picca et al., 2019a, 2020a) to simultaneously analyze biomediators pertaining to three different domains: inflammation, amino acid metabolism, and mitochondrial quality control (MQC). The availability of systemic inflammatory and metabolic data from this cohort (Calvani et al., 2018b; Marzetti et al., 2019) and their complementation with the analysis of circulating MDVs (Picca et al., 2020a) provided a composite dataset to explore the relationship among systemic inflammation, metabolic characteristics, and MDV trafficking in PF&S. Data analysis was performed through sequential and orthogonalized covariance selection coupled with linear discriminant analysis (SO-CovSel–LDA), an innovative analytical strategy that is particularly suited for dealing with multi-block datasets (i.e., experimental settings in which variables are assayed using different platforms and/or at different time points) (Biancolillo et al., 2020). SO-CovSel–LDA enabled selecting the variables of interest for PF&S from a large number of highly correlated candidate biomarkers. The evaluation of these biomarkers in larger cohorts and their changes over time or in response to interventions may unveil specific pathophysiological pathways of PF&S and identify biological targets for drug development.

## Materials and Methods

### Participants

Participants of the present study were community-dwellers aged 70+ with PF&S and non-sarcopenic non-frail (nonPF&S) controls from the BIOSPHERE cohort (Calvani et al., 2018c). BIOSPHERE is a cross-sectional study conceived for selecting and validating a panel of candidate biomarkers for PF&S through multivariate statistical modeling (Calvani et al., 2018b,c; Picca et al., 2019a).

To diagnose PF&S, the operational definition developed in the “Sarcopenia and Physical fRailty IN older people: multi-componenT Treatment strategies” (SPRINTT) project (Marzetti et al., 2015, 2018) was applied: (a) physical frailty, based on a summary score on the Short Physical Performance Battery (SPPB) (Guralnik et al., 1994) between 3 and 9; (b) low appendicular muscle mass (aLM), according to the cut-points of the Foundation for the National Institutes of Health Sarcopenia Project (Studenski et al., 2014); and (c) absence of major mobility disability (i.e., inability to complete a 400-m walk test) (Newman et al., 2006). Data analysis for the present investigation was conducted in a convenience sample of 21 participants (11 older adults with PF&S and 10 nonPF&S controls) for whom complete data were available for inflammatory and metabolic mediators and MDV characterization.

The Ethics Committee of the Università Cattolica del Sacro Cuore (Rome, Italy; protocol number BIOSPHERE: 8498/15) approved the study protocol. All procedures were conducted in compliance to the ethical standards laid down in the 1964 Declaration of Helsinki and its later amendments. Study procedures and criteria for participant selection were described thoroughly elsewhere (Calvani et al., 2018c). A written inform consent form was signed by all participants prior to enrolment.

### Assessment of Appendicular Lean Mass by Dual X-Ray Absorptiometry

Appendicular lean mass was quantified through whole-body dual X-ray absorptiometry scans on a Hologic Discovery A densitometer (Hologic, Inc., Bedford, MA, USA) according to the manufacturer's directions. Criteria for low aLM were as follows: (a) aLM to body mass index (BMI) ratio (aLM_BMI_) <0.789 and <0.512 in men and women, respectively; or (b) crude aLM <19.75 kg in men and <15.02 kg in women when the aLM_BMI_ criterion was not met (Studenski et al., 2014).

### Collection and Processing of Blood Samples

Blood samples were obtained in the morning after overnight fasting by venipuncture of the median cubital vein, using BD Vacutainer^®^ commercial tubes (Becton, Dickinson and Co., Franklin Lakes, NJ, USA). One tube was delivered to the centralized diagnostic laboratory of the Fondazione Policlinico Universitario “Agostino Gemelli” IRCCS (Rome, Italy) for standard blood biochemistry. The remaining tubes were processed for serum separation in the Biogerontology lab of the Università Cattolica del Sacro Cuore. After 30 min of blood clotting at room temperature, samples were centrifuged at 1,000 × *g* for 15 min at 4°C. The upper clear fraction (serum) was recovered in 0.5-mL aliquots and stored at −80°C until analysis.

### Purification of Small Extracellular Vesicles From Serum

Small EVs were purified from serum through differential centrifugation and were quantified as previously reported (Picca et al., 2018a, 2019b, 2020a). In brief, serum samples were diluted with equal volumes of phosphate-buffered saline (PBS) and centrifuged at 2,000 × *g* at 4°C for 30 min. Supernatants were collected and centrifuged at 12,000 × *g* at 4°C for 45 min to remove apoptotic bodies, mitochondrial fragments, cell debris, and vesicles larger than 200 nm. Supernatants were subsequently ultracentrifuged at 110,000 × *g* at 4°C for 2 h. Pellets were recovered and resuspended in PBS, filtered through a 0.22-μm filter and ultracentrifuged at 110,000 × *g* at 4°C for 70 min to eliminate contaminant proteins. Pellets enriched in purified sEVs were finally resuspended in 100 μL of PBS. To quantify sEVs, total protein concentration was measured using the Bradford assay (Théry et al., 2006).

### Measurement of Inflammatory, Metabolic, and Mitochondrial Markers

Serum samples were assayed for a panel of 75 candidate biomarkers (Table 1). The panel was designed based on previous studies in older adults and their involvement in pathways and processes relevant to PF&S pathophysiology (i.e., inflammation, amino acid metabolism, and mitochondrial dysfunction) (Calvani et al., 2018b, 2020a; Marzetti et al., 2019; Picca et al., 2019a, 2020b).

**Table 1:** Composition of the biomarker panel.

**Biological pathways**	**Biomolecules**
Inflammation	BDNF, CRP, IL1-β, IL1-ra, IL2, IL4, IL5, IL6, IL7, IL8, IL9, IL10, IL12, IL13, IL15, IL17, FGF basic, FGF21, G-CSF, GM-CSF, IFN-γ, MCP-1, MIP-1α, MIP-1β, CCL5, CCL11, IP-10, MPO, PDGF-BB, TNF-α, VEGF
Amino acid metabolism	1-methylhistidine, 3-methylhistidine, 4-hydroxyproline, α-aminobutyric acid, β-alanine, β-aminobutyric acid, γ-aminobutyric acid, alanine, aminoadipic acid, anserine, arginine, asparagine, aspartic acid, carnosine, citrulline, cystathionine, cystine, ethanolamine, glutamic acid, glycine, histidine, isoleucine, leucine, lysine, methionine, ornithine, phenylalanine, phosphoethanolamine, phosphoserine, proline, sarcosine, serine, taurine, threonine, tryptophan, tyrosine, valine
MDVs	ATP5A, CD63, MTCOI, NDUFB8, NDUFS3, SDHB, UQCRC2

*ATP5A, adenosine triphosphate 5A; BDNF, brain-derived neurotrophic factor; CCL, C-C motif chemokine ligand; CD, cluster of differentiation; CRP, C-reactive protein; FGF, fibroblast growth factor; G-CSF, granulocyte colony-stimulating factor; GM-CSF, granulocyte-macrophage colony-stimulating factor; IFN, interferon; IL, interleukin; IL1-ra, IL1 receptor agonist; IP, IFN-α-induced protein; MCP-1, monocyte chemoattractant protein 1; MDVs, mitochondrial-derived vesicles; MIP-1α, macrophage inflammatory protein 1α; MIP-1β, macrophage inflammatory protein 1β; MPO, myeloperoxidase; MTCOI, mitochondrial cytochrome C oxidase subunit I; NDUFB8, nicotinamide adenine dinucleotide reduced form (NADH):ubiquinone oxidoreductase subunit B8; NDUFS3, NADH:ubiquinone oxidoreductase subunit S3; PDGF-BB, platelet-derived growth factor BB; SDHB, succinate dehydrogenase complex iron sulfur subunit B; TNF-α, tumor necrosis factor alpha; UQCRC2, ubiquinol-cytochrome C reductase core protein 2; VEGF, vascular endothelial growth factor*.

Twenty-seven inflammatory mediators including cytokines, chemokines, and growth factors were assayed using the Bio-Plex Pro™ Human Cytokine 27-plex Assay kit (#M500KCAF0Y, Bio-Rad Laboratories Inc., Hercules, CA, USA) on a Bio-Plex^®^ System with Luminex xMAP^®^ Technology (Bio-Rad Laboratories), as described elsewhere (Ponziani et al., 2018, 2019; Marzetti et al., 2019; Picca et al., 2019a; Addolorato et al., 2020). Serum levels of C-reactive protein (CRP), myeloperoxidase (MPO), fibroblast growth factor (FGF) 21, and brain-derived neurotrophic factor (BDNF) were measured by using commercially available kits on an ELLA™ automated immunoassay system (Bio-Techne, San Jose, CA, USA). The concentration of 37 amino acids and derivatives was determined by ultraperformance liquid chromatography/mass spectrometry (UPLC/MS) as described previously (Calvani et al., 2018b).

Protein levels of tetraspanin CD63 and selected mitochondrial markers were measured by Western immunoblot analysis in purified sEVs, as detailed elsewhere (Picca et al., 2020a). According to the guidelines of the International Society of Extracellular Vesicles (Théry et al., 2018), sEV purity was ascertained by verifying the presence of the cytosolic protein flotilin (positive control) and the absence of the non-sEV component heterogeneous nuclear ribonucleoprotein A1 (HNRNPA1, negative control). Scanning electron microscopy analyses were performed to confirm enrichment in sEVs (Théry et al., 2018).

### Statistical Analysis

#### Descriptive Statistics

Descriptive statistics were run on all data. The normal distribution of data was ascertained via the Kolmogorov–Smirnov test. Differences in demographic, anthropometric, clinical, and biological parameters between participants with and without PF&S were assessed via *t*-test statistics or the Mann–Whitney *U*-test, as appropriate, for continuous variables. Chi-squared χ^2^ or Fisher exact tests were applied for categorical variables. All tests were two-sided, with statistical significance set at *p* < 0.05. Analyses were performed using the GraphPrism 5.03 software (GraphPad Software, Inc., San Diego, CA, USA).

#### Sequential and Orthogonalized Covariance Selection – Linear Discriminant Analysis

In order to identify relationships among different sets of data and to identify putative markers of PF&S, a recently proposed multi-block classification approach, called SO-CovSel–LDA (Biancolillo et al., 2020), was adopted. SO-CovSel–LDA is a highly efficient multi-block classification strategy, which combines a very parsimonious variable selection algorithm to be applied on each individual block (CovSel) (Roger et al., 2011) with the sequential inclusion of data matrices, after orthogonalization with respect to the previously selected variables. This procedure reduces redundancies among the blocks and allows a clearer interpretation of results.

Here, we begin with describing the CovSel variable selection algorithm for a single block of predictors **X** to make its generalization to the case of multiple blocks easier to follow. CovSel allows selecting the minimum number of variables that provide an accurate regression model between the **X** matrix and a response **Y**. Variables are progressively selected from the **X**-block as those having maximum covariance with **Y**. Parsimony is achieved as any successive predictor is selected after both **X** and **Y** are orthogonalized with respect to the previously chosen variables, so to bring as much new information as possible. SO-CovSel is a generalization of this procedure to a multi-block case, enriched by the concept of sequential inclusion of the blocks after orthogonalization which is borrowed from methods such as SO partial least squares (SO-PLS) regression (Biancolillo and Næs, 2019; Biancolillo et al., 2019). In the case of three blocks of predictors, like in the present study (i.e., inflammatory markers, amino acids, and MDV cargo molecules), the SO-CovSel algorithm proceeds as follows. Variables are selected from the first block (amino acids and derivatives) using CovSel, then both the second block (inflammatory markers) **X**_2_ and the **Y** are orthogonalized with respect to the variables selected from the first block (**X**_1,sel_):

(1) $$\begin{array}{l} {\text{X}}_{2,\;orth}=\left[\text{I}-{\text{X}}_{1,sel}{\left({\text{X}}_{1,\;sel}^{T}{\text{X}}_{1,sel}\right)}^{-1}{\text{X}}_{1,\;sel}^{T}\right]{\text{X}}_{2} \end{array}$$

(2) $$\begin{array}{l} {\text{Y}}_{orth}=\left[\text{I}-{\text{X}}_{1,sel}{\left({\text{X}}_{1,\;sel}^{T}{\text{X}}_{1,sel}\right)}^{-1}{\text{X}}_{1,\;sel}^{T}\right]\text{Y} \end{array}$$

where **I** is the identity matrix and the superscript *T* indicates matrix transposition. CovSel is then applied to the matrix **X**_2,*orth*_ to select variables having maximum covariance with **Y**_*orth*_; the selected variables are subsequently collected in the matrix **X**_2,*sel*_. The third block (MDV markers) and the **Y** are orthogonalized with respect to the variables selected from the first two blocks, similar to what described in equations (1) and (2). CovSel is applied to the orthogonalized third block **X**_3,*orth*_ to select variables (**X**_3,*sel*_) with maximum covariance with the orthogonalized response. Finally, an overall regression model is built between the selected variables from the three blocks and the **Y**:

(3) $$\begin{array}{l} \hat{\text{Y}}={{\text{X}}_{1,sel}\text{B}}_{1}+{{\text{X}}_{2,sel}\text{B}}_{2}+{{\text{X}}_{3,sel}\text{B}}_{3}\;\text{Y} \end{array}$$

where $\hat{\text{Y}}\;$is the predicted response and **B**_1_, **B**_2_, and **B**_3_ are the regression coefficients.

SO-CovSel–LDA is the classification analog of the SO-CovSel regression algorithm. As such, it requires the class information to be encoded in a binary-coded response vector **y**. The response vector will have the value 1 for PF&S participants and 0 for nonPF&S controls. A SO-CovSel model is built between the different blocks of predictors and the **y** as described above. Eventually, LDA is applied to the predicted response to accomplish sample classification.

To unbiasedly validate the results, a repeated double cross-validation (rDCV) procedure was used (Smit et al., 2007; Biancolillo et al., 2019). DCV consists of two loops of cross-validation nested into one another: the inner loop is used for model selection and the outer loop for external validation (i.e., contains samples that were not use at any stage of model building or optimization). Since DCV implies splitting the samples into the different cross-validation groups, to avoid the outcomes depending on a particular splitting scheme, the whole procedure is repeated a certain number of times (50 in the present study). In the context of biomarker discovery, the use of rDCV has the advantage that many different models are calculated (as many as the product of the number of DCV runs times the number of splits in the inner loop), which enables evaluating how consistently variables are selected by SO-CovSel–LDA and how robust candidate markers are. All calculations were carried out using in-house written functions running under Matlab environment (R2015b, The Mathworks, Natick, MA) and freely downloadable at: https://www.chem.uniroma1.it/romechemometrics/research/algorithms/so-covsel/.

## Results

### Characteristics of the Study Participants

Data from 21 BIOSPHERE participants, 11 older adults with PF&S (mean age 77.7 ± 5.4 years; 73.0% women) and 10 nonPF&S controls (mean age 73.9 ± 2.7 years; 50.0% women) were analyzed in the present study. Demographic, functional, anthropometric, and clinical characteristics of the included participants were comparable to those of the whole BIOSPHERE cohort (Marzetti et al., 2019).

As shown in Table 2, no differences were observed in sex distribution, BMI, or number of comorbid conditions and medications between PF&S and controls participants. The latter group was slightly younger, but the age difference did not reach statistical significance. Serum albumin, total serum protein concentration, and the neutrophil count were comparable between groups. On the other hand, the lymphocyte count was lower and the neutrophil-to-lymphocyte ratio was higher in the PF&S group. As per the inclusion criteria adopted, participants with PF&S had lower SPPB scores and smaller aLM either crude or adjusted by BMI compared with nonPF&S controls.

**Table 2:** Participant characteristics according to the presence of physical frailty & sarcopenia.

**Characteristic**	**nonPF&S (*n* = 10)**	**PF&S (*n* = 11)**	***p*-value**
Age (years), mean ± SD	73.9 ± 2.7	77.7 ± 5.4	0.0557
Gender (female), *n* (%)	5 (50)	8 (73)	0.5344
BMI (kg/m^2^), mean ± SD	28.1 ± 2.8	30.3 ± 4.3	0.1891
SPPB summary score, mean ± SD	12.0 ± 1.0	7.0 ± 0.3	<0.0001
aLM (kg), mean ± SD	20.21 ± 4.10	15.84 ± 3.63	0.0390
aLM_BMI_, mean ± SD	0.81 ± 0.32	0.51 ± 0.11	0.0118
Albumin (g/L), mean ± SD	45.4 ± 12.7	39.8 ± 1.2	0.1536
Total serum protein concentration (g/L), mean ± SD	71.8 ± 4.6	75.5 ± 3.1	0.0914
Neutrophil count (10^9^/L)	3.46 ± 1.40	4.07 ± 1.10	0.2757
Lymphocyte count (10^9^/L)	1.76 ± 0.41	1.37 ± 0.30	0.0222
Neutrophil/lymphocyte	1.94 ± 0.57	3.13 ± 1.14	0.0076
Number of diseases^*^, mean ± SD	3.2 ± 1.6	3.1 ± 1.2	0.8647
Number of medications^§^, mean ± SD	2.9 ± 1.6	3.2 ± 1.8	0.7061

*aLM, appendicular lean mass; aLM_BMI_, aLM adjusted by body mass index (BMI); nonPF&S, non-physically frail, non-sarcopenic; PF&S, physical frailty & sarcopenia; SD, standard deviation*.

* *Includes hypertension, coronary artery disease, prior stroke, peripheral vascular disease, diabetes, chronic obstructive pulmonary disease, and osteoarthritis*.

§ *Includes prescription and over-the-counter medications*.

### Biomarker Selection Through SO-CovSel–LDA Analysis

Serum levels of 75 inflammatory cytokines, growth factors, neurogenesis and neural plasticity mediators, amino acids and derivatives, and MDV cargo molecules were measured through multiple analytical platforms. Of the assayed biomolecules, concentrations of anserine, carnosine, cystathionine, γ-aminobutyric acid, phosphoserine, interleukin (IL) 2, IL5, IL7, IL10, IL13, IL15, granulocyte colony-stimulating factor, vascular endothelial growth factor, mitochondrial cytochrome C oxidase subunit I, nicotinamide adenine dinucleotide reduced form (NADH):ubiquinone oxidoreductase subunit B8, and ubiquinol-cytochrome C reductase core protein 2 were below the detection limit. Serum concentrations of the assayed biomolecules are shown in Supplementary Table 1. SO-CovSel–LDA models were built using a multi-matrix dataset containing 59 analytes. Serum concentrations of candidate biomarkers were organized into three matrices according to the analytical approach adopted for their determination and the biological domain of pertinence (Table 1). Prior to data analysis, blocks were individually pretreated by autoscaling through subtracting from each variable its mean and dividing the result by its standard deviation. Afterwards, SO-CovSel–LDA models were built and validated via 50 runs of rDCV with 21 cancelation groups in the outer loop and five in the inner loop. The optimal order of blocks and the optimal number of variables from each block were selected as those returning the smallest error in the inner cross-validation loop. Since a total of 50 × 21 (i.e., 1,050) different models were built, the procedure allowed calculating the confidence intervals for the predictive ability and the consistency of selection of the optimal number of predictors as well as revealing their identity.

The predictive ability was evaluated on the samples of the outer rDCV loop, since they were completely external of the models used for their prediction, thereby providing more unbiased estimates of the discriminant capacity. Our analytical approach was able to correctly classify 87.5 ± 7.3% of PF&S participants and 83.6 ± 9.2% of nonPF&S controls, corresponding to an overall classification accuracy of 85.6 ± 5.0%. Permutation tests with 1,000 randomizations, which describe the distribution of figures of merit under the null hypothesis, showed that the discriminant ability of the model was statistically significant (*p* < 0.001).

**Figure 1:**
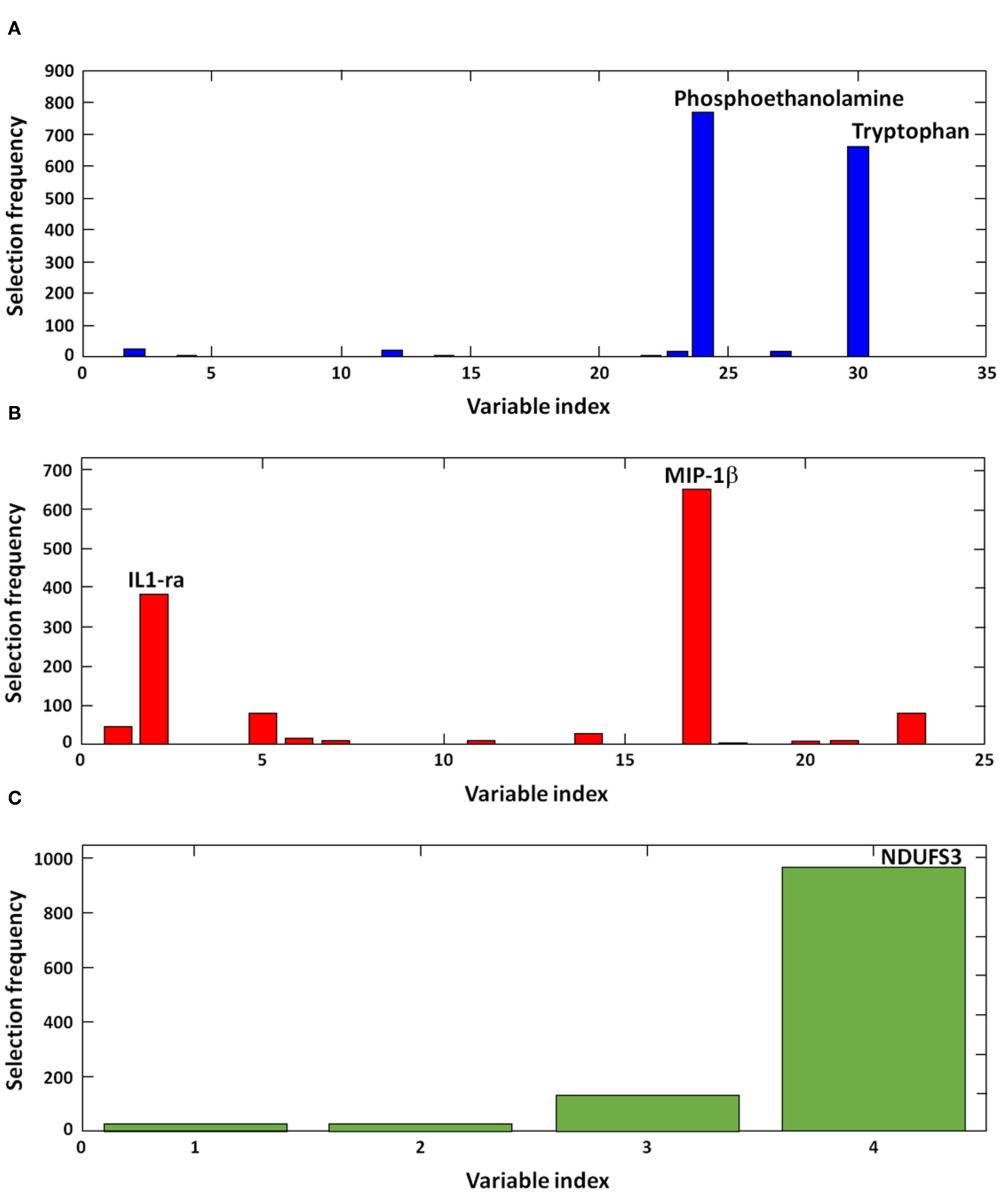
Discriminant biomolecules selected by sequential and orthogonalized covariance selection coupled with linear discriminant analysis (SO-CovSel–LDA). Candidate biomarkers are presented according to the order in which they respective data matrices were entered into the model: **(A)** amino acids and derivatives, **(B)** inflammatory biomolecules, and **(C)** mitochondrial-derived vesicle cargo molecules. IL1-ra, interleukin 1 receptor agonist; MIP-1β, macrophage inflammatory protein 1β; NDFUS3, nicotinamide adenine dinucleotide reduced form (NADH), ubiquinone oxidoreductase subunit S3.

Results appeared to be highly consistent across rDCV runs. Indeed, in all of the 1,050 models calculated, the best order of the blocks was found to be (1) amino acids and derivatives, (2) inflammatory biomolecules, and (3) MDV markers. In the large majority of iterations, the optimal model complexity was found to be 2, 2, and 1 variables, respectively. Figure 1 illustrates the frequency of selection of discriminant markers across the 1,050 models calculated during the rDCV procedure. The figure shows that the optimal model complexity was consistently found to be 2-2-1, and that the same biomolecules—i.e., phosphoethanolamine, tryptophan, IL1 receptor antagonist (IL1-ra), macrophage inflammatory protein 1β (MIP-1β), and NDUF subunit S3 (NDUFS3)—were selected in almost all iterations, thus confirming the robustness of the proposed biomarkers in our sample.

## Discussion

According to the geroscience paradigm, the roots of most chronic diseases reside in perturbations of a discrete set of biological mechanisms, collectively termed hallmarks of aging (López-Otín et al., 2013). The pathophysiology of sarcopenia involves all major biological pillars of aging and is, therefore, envisioned as a prototypical geroscience condition (Calvani et al., 2020a). In particular, derangements of skeletal myocyte quality control mechanisms are thought to play a relevant role in the development and progression of age-related muscle wasting (Iqbal et al., 2013). Indeed, altered recycling of damaged cell components and organelles via autophagy, defective mitochondrial proteostasis and dynamics, and impaired mitochondriogenesis have been described in muscles of older adults with PF&S (Picca et al., 2018a). Systemic signatures of PF&S, including specific inflammatory and amino acid profiles, have also been identified (Calvani et al., 2018b, 2020a; Marzetti et al., 2019).

In the present study, we applied an innovative SO-CovSel–LDA analytical approach to explore the relationship among systemic inflammation, metabolic derangements, and circulating MDVs in PF&S in order to provide hints on the underlying pathogenic mechanisms. Among all assayed molecules, SO-CovSel–LDA selected a panel comprising two amino acids (i.e., phosphoethanolamine and tryptophan), two cytokines (i.e., IL1-ra and MIP-1β), and NDUFS3, a subunit of complex I of the mitochondrial electron transport chain (ETC), as the best predictors to discriminate older adults with and without PF&S.

The identification of phosphoethanolamine within the discriminant metabolites for the classification of PF&S is particularly relevant. Phosphoethanolamine is an ethanolamine derivate produced as an intermediate of the CDP-ethanolamine pathway involved in the metabolism of glycerophospholipid and biological membrane turnover (Patel and Witt, 2017; van der Veen et al., 2017). Disruption of CDP-ethanolamine pathway has been associated with mitochondrial dyshomeostasis in mouse models of muscle atrophy (Selathurai et al., 2019) and insulin resistance (Funai et al., 2016). Phosphoethanolamine has also been found to mediate mitochondrial membrane fusion and curvatures and, in combination with ethanolamine, to promote autophagy and longevity (Rockenfeller et al., 2015). The difference in phosphoethanolamine serum levels between older adults with and without PF&S might reflect impairment of autophagy in the setting of muscle atrophy (Iqbal et al., 2013; Marzetti et al., 2016). This hypothesis is in keeping with the higher secretion of sEVs previously described in older adults with PF&S (Picca et al., 2020a), which could be interpreted as an attempt to cope with deficient MQC processes (Soubannier et al., 2012; Picca et al., 2020b). Noticeably, derangements in the MQC machinery were documented in intraoperative muscle biopsies obtained from old hip-fractured patients with sarcopenia (Marzetti et al., 2016). Finally, phosphoethanolamine was also found among the mediators possibly involved in the disabling cascade in frail older persons with type 2 diabetes mellitus (T2DM) (Calvani et al., 2020b).

The amino acidic profile of participants with PF&S also included the aromatic essential amino acid tryptophan. The latter regulates several activities within the body, including growth, mood, behavior, and immune responses (Le Floc'h et al., 2011). Its metabolism is mediated by the tryptophan-kynurenine and the tryptophan-methoxyndole pathways, leading to the production of physiologically relevant bioactive compounds, such as NAD, serotonin, and melatonin (Le Floc'h et al., 2011). Changes in tryptophan circulating levels have been associated with low muscle quality (Moaddel et al., 2016), insulin resistance, and frailty in older adults with T2DM (Chen et al., 2016; Marcos-Pérez et al., 2017; Calvani et al., 2020b).

As per the inflammatory fingerprint of PF&S, IL1-ra and MIP-1β were selected by SO-CovSel–LDA as the most relevant mediators. A frailty “cytokinome” in older adults with PF&S composed of a “core” inflammatory profile with gender-specific signatures was previously described by our group (Marzetti et al., 2019). In a later study, SO-CovSel allowed restricting to only MPO and platelet-derived growth factor BB the mediators describing the contribution of inflammation to PF&S (Calvani et al., 2020a). In the same study, gender-specific models selected MIP-1β as one of the most relevant biomarkers for the discrimination between PF&S and nonPF&S participants (Calvani et al., 2020a). MIP-1β is a chemokine that regulates myoblast response to muscle injury and promotes leucocyte recruitment at the site of muscle damage (Yahiaoui et al., 2008). The presence of MIP-1β among discriminant analytes for PF&S may be interpreted as a compensatory action to impaired muscle regenerative capacity (Calvani et al., 2020a). Inflamm-aging, the chronic low-grade inflammation that develops during aging, has been involved in the pathogenesis of sarcopenia and physical disability (Wilson et al., 2017; Franceschi et al., 2018; Furman et al., 2019). In this context can be framed the selection of IL1-ra among the discriminant biomolecules associated with PF&S. IL1-ra is a natural negative modulator of IL1α- and IL1β-mediated inflammatory response and was found to be overexpressed in muscles of older adults (Przybyla et al., 2006). The inflammatory milieu of PF&S is also reflected by the higher neutrophil-to-lymphocyte ratio, an easily accessible indicator of inflammation.

Finally, the MDV cargo molecule NDUFS3 was selected among the discriminant mediators for PF&S. NDUFS3 is a nuclear-encoded subunit of complex I of the mitochondrial ETC. Mutations of NDUFS3 are associated with deficiency in mitochondrial respiration and myopathies (Bénit et al., 2004; Pereira et al., 2020). Conversely, NDUFS3 gene replacement rescued muscle structure and mitochondrial in a mouse model of mitochondrial myopathy (Pereira et al., 2020). Mitochondrial dysfunction and insufficient MDV-mediated quality control have also been hypothesized to contribute to PF&S (Picca et al., 2020a). This idea is in keeping with the altered expression of key MQC proteins described in muscles of old hip-fractured patients with sarcopenia (Marzetti et al., 2016).

Taken as a whole, our findings provide preliminary, yet novel insights into the relationship among metabolic changes, inflamm-aging, and mitochondrial dyshomeostasis in PF&S. In particular, the retrieval of MDVs in serum of older adults with PF&S allows placing this process in the context of an innate immune response as part of the “danger theory” of inflammation (Zhang et al., 2010). According to this view, MDVs may function as antigen-presenting vesicles carrying misplaced noxious material. Similar to damage-associated molecular patterns (DAMPs) released from injured cells, the MDV cargo can trigger caspase-1 activation and the secretion of pro-inflammatory cytokines (Krysko et al., 2011). This inflammatory response would be mounted through the interactions of mitochondrial DAMPs with receptors/systems including Toll-like receptors, Nod-like receptor family pyrin domain containing 3 inflammasome, and cytosolic cyclic GMP–AMP synthase-stimulator of interferon genes DNA sensing system (Picca et al., 2017b). Impaired MQC in skeletal myocytes may therefore install a vicious circle favoring further mitochondrial damage and the propagation of sterile inflammation through DAMPs release (Picca et al., 2018b). Should this hypothesis be confirmed, the scavenging of circulating mitochondrial DAMPs might be exploited for the development of therapeutic interventions for PF&S.

Albeit presenting promising findings, our work has limitations that deserve discussion. First of all, the study was conducted in a small participant sample and results need to be validated in larger cohorts. Although participants were carefully selected and thoroughly characterized, the chance of unknown comorbidities affecting the results cannot be excluded. Also, the cross-sectional design of the study hampers establishing cause-effect or temporal relationships between the analyzed pathways and PF&S development. Finally, while the biomarker panel analyzed in the present study included a large number of biomolecules, the possibility exists that a more comprehensive appraisal of PF&S pathophysiology might be obtained through the analysis of larger sets of mediators.

## Data Availability Statement

All datasets generated for this study are included in the article/Supplementary Material.

## Ethics Statement

The studies involving human participants were reviewed and approved by Università Cattolica del Sacro Cuore, Rome, Italy. The participants provided their written informed consent to participate in this study.

## Author Contributions

APi, CB, EM, FG, and RC: conceptualization. APi and FG: data curation. AB and FM: data analysis. APi, APr, FG, HC-J, and JG: methodology. APi, EM, and RC: writing—original draft preparation. CB, FG, and FL: writing—review and editing. FL and RB: supervision. CB and RB: funding acquisition. All authors contributed to the article and approved the submitted version.

## Conflict of Interest

EM served as a consultant for Abbott, Biophytis, Nutricia, and Nestlè. RC served as a consultant for Abbott and Nutricia. The remaining authors declare that the research was conducted in the absence of any commercial or financial relationships that could be construed as a potential conflict of interest. The handling editor declared a past co-authorship with several of the authors EM, RC, and AP. The reviewers FS and EP declared a past collaboration with one of the authors EM to the handling editor.
