# Supplementary material for: Circulating Mitochondrial-Derived Vesicles, Inflammatory Biomarkers and Amino Acids in Older Adults With Physical Frailty and Sarcopenia: A Preliminary BIOSPHERE Multi-Marker Study Using Sequential and Orthogonalized Covariance Selection – Linear Discriminant Analysis
Source: Front Cell Dev Biol. 2020 Sep 22;8:564417. doi: 10.3389/fcell.2020.564417 (PMC7536309; doi:10.3389/fcell.2020.564417)
Supplement: Supplementary file 1 [file Table_1.DOCX]

**Supplementary table 1.** Serum concentrations of inflammatory mediators and amino acid and derivatives, and quantification of selected mitochondrial-derived vesicle markers in participants with and without physical frailty & sarcopenia.

| **Biological pathways** | **Assayed biomolecules** | **nonPF&S (*n* = 10)** | **PF&S (*n* = 11)** | **p** |
| --- | --- | --- | --- | --- |
| Inflammation | BDNF (ng/mL) | 31.47 ± 14.35 | 36.89 ± 9.38 | 0.3134 |
|  | CCL11 (pg/mL) | 230.96 ± 118.98 | 175.34 ± 93.35 | 0.2456 |
|  | CCL5 (μg/mL) | 7.83 ± 7.71 | 1.51 ± 3.45 | 0.0529 |
|  | CRP (μg/mL) | 0.74 ± 0.44 | 0.32 ± 0.19 | 0.0184 |
|  | FGF basic (pg/mL) | 36.87 ± 13.20 | 53.55 ± 8.96 | 0.0028 |
|  | FGF21 (pg/mL) | 480.85 ± 500.51 | 426.34 ± 133.24 | 0.1489 |
|  | GM-CSF (pg/mL) | 1.61 ± 0.59 | 1.00 ± 0.56 | 0.0248 |
|  | IFNγ (pg/mL) | 2.69 ± 0.61 | 3.43 ± 1.28 | 0.0974 |
|  | IL12 (pg/mL) | 2.12 ± 1.49 | 2.91 ± 1.01 | 0.3404 |
|  | IL17 (pg/mL) | 16.16 ± 6.31 | 24.20 ± 8.95 | 0.0293 |
|  | IL1ra (pg/mL) | 125.65 ± 40.73 | 165.54 ± 28.48 | 0.0167 |
|  | IL1ß (pg/mL) | 1.11 ± 0.34 | 0.96 ± 0.35 | 0.3295 |
|  | IL4 (pg/mL) | 2.09 ± 2.07 | 5.23 ± 2.48 | 0.0054 |
|  | IL6 (pg/mL) | 2.73 ± 0.64 | 2.67 ± 1.35 | 0.8978 |
|  | IL8 (pg/mL) | 12.63 ± 7.32 | 14.06 ± 3.56 | 0.5696 |
|  | IL9 (pg/mL) | 123.60 ± 49.65 | 115.18 ± 16.06 | 0.5974 |
|  | IP10 (pg/mL) | 934.40 ± 648.32 | 883.98 ± 702.04 | 0.6472 |
|  | MCP1 (pg/mL) | 33.65 ± 12.54 | 32.88 ± 19.17 | 0.9160 |
|  | MIP-1α (pg/mL) | 9.04 ± 5.32 | 3.56 ± 1.79 | 0.0265 |
|  | MIP-1β (pg/mL) | 137.00 ± 54.69 | 198.63 ± 23.50 | 0.0029 |
|  | MPO (μg/mL) | 5.911 ± 10.82 | 23.98 ± 27.05 | 0.6986 |
|  | PDGF-BB (μg/mL) | 3.29 ± 1.38 | 3.49 ± 1.49 | 0.7483 |
|  | TNF-α (pg/mL) | 31.69 ± 16.22 | 44.66 ± 10.19 | 0.0390 |
| Amino acid metabolism | α-amino butyric acid (µmol/L) | 26.16 ± 6.14 | 20.92 ± 9.55 | 0.1558 |
|  | β-alanine (µmol/L) | 6.28 ± 3.04 | 5.39 ± 2.63 | 0.4927 |
|  | β-amino butyric acid (µmol/L) | 2.42 ± 1.52 | 2.33 ± 0.83 | 0.8699 |
|  | 1-methylhistidine (µmol/L) | 6.74 ± 7.52 | 2.64 ± 1.49 | 0.2905 |
|  | 3-methylhistidine (µmol/L) | 4.52 ± 1.55 | 5.01 ± 1.87 | 0.5972 |
|  | 4-hydroxyproline (µmol/L) | 18.18 ± 9.35 | 15.38 ± 5.57 | 0.4097 |
|  | Alanine (µmol/L) | 459.45 ± 102.18 | 470.43 ± 145.67 | 0.8452 |
|  | Aminoadipic acid (µmol/L) | 1.82 ± 0.95 | 1.95 ± 0.88 | 0.4590 |
|  | Arginine (µmol/L) | 114.23 ± 45.05 | 95.77 ± 28.31 | 0.2453 |
|  | Asparagine (µmol/L) | 105.23 ± 27.77 | 99.89 ± 27.29 | 0.6620 |
|  | Aspartic acid (µmol/L) | 22.12 ± 3.54 | 18.25 ± 3.28 | 0.0174 |
|  | Citrulline (µmol/L) | 39.18 ± 13.23 | 34.26 ± 10.13 | 0.3474 |
|  | Cystine (µmol/L) | 24.14 ± 8.59 | 30.05 ± 9.64 | 0.1562 |
|  | Ethanolamine (µmol/L) | 10.08 ± 3.03 | 10.52 ± 2.08 | 0.7011 |
|  | Glycine (µmol/L) | 263.53 ± 63.49 | 279.97 ± 78.27 | 0.6055 |
|  | Glutamic acid (µmol/L) | 93.64 ± 39.49 | 86.22 ± 30.35 | 0.6327 |
|  | Histidine (µmol/L) | 87.57 ± 11.38 | 91.62 ± 12.78 | 0.4546 |
|  | Isoleucine (µmol/L) | 76.02 ± 24.22 | 68.66 ± 24.39 | 0.4969 |
|  | Leucine (µmol/L) | 114.70 ± 38.66 | 142.73 ± 39.80 | 0.8193 |
|  | Lysine (µmol/L) | 219.64 ± 51.60 | 201.59 ± 29.97 | 0.8603 |
|  | Methionine (µmol/L) | 26.16 ± 8.33 | 24.69 ± 4.49 | 0.6159 |
|  | Ornithine (µmol/L) | 119.98 ± 28.81 | 108.40 ± 34.02 | 0.4129 |
|  | Phenylalanine (µmol/L) | 81.27 ±11.90 | 73.28 ± 7.86 | 0.0826 |
|  | Phosphoethanolamine (µmol/L) | 1.24 ± 0.40 | 2.45 ± 1.13 | 0.0043 |
|  | Proline (µmol/L) | 259.99 ± 84.33 | 258.70 ± 127.04 | 0.9786 |
|  | Sarcosine (µmol/L) | 1.94 ± 1.31 | 1.95 ± 0.51 | 0.4593 |
|  | Serine (µmol/L) | 128.48 ± 15.25 | 140.07 ± 20.27 | 0.1583 |
|  | Taurine (µmol/L) | 181.49 ± 46.60 | 192.88 ± 59.28 | 0.6325 |
|  | Threonine (µmol/L) | 137.95 ± 32.65 | 128.61 ± 28.81 | 0.4944 |
|  | Tryptophan (µmol/L) | 67.38 ± 10.91 | 55.13 ± 5.04 | 0.0033 |
|  | Tyrosine (µmol/L) | 73.33 ± 17.52 | 65.65 ± 13.14 | 0.2673 |
|  | Valine (µmol/L) | 253.10 ± 68.09 | 250.33 ± 67.21 | 0.9262 |
| MDVs | ATP5A (AU) | 32.83 ± 50.54 | 60.64 ± 34.32 | 0.2175 |
|  | CD63 (AU) | 27.85 ± 31.82 | 8.95 ± 6.21 | 0.2597 |
|  | NDUFS3 (AU) | 52.17 ± 42.81 | 14.95 ± 11.58 | 0.0184 |
|  | SDHB (AU) | 89.49 ± 164.84 | 18.53 ± 11.49 | 0.2179 |

Data are shown as mean ± standard deviation.

*Abbreviations*: ATP5A: adenosine triphosphate 5A; AU: arbitrary unit; BDNF: brain-derived neutrophic factor; CCL, C-C motif chemokine ligand; CD: cluster of differentiation; CRP: C-reactive protein; FGF: fibroblast growth factor; GM-CSF: granulocyte-macrophage colony-stimulating factor; IFN: interferon; IL: interleukin; IL1ra, interleukin 1 receptor agonist; IP: interferon gamma-induced protein; MCP1: monocyte chemoattractant protein 1; MDVs: mitochondrial-derived vesicles; MIP: macrophage inflammatory protein; MPO: myeloperoxidase; NDUFS3: nicotinamide adenine dinucleotide reduced form:ubiquinone oxidoreductase subunit S3; PDGF-BB, platelet derived growth factor BB; PF&S: physical frailty & sarcopenia; SDHB: succinate dehydrogenase complex iron sulfur subunit B; TNF, tumor necrosis factor.
